# From The Editor’s Desk

**Published:** 2018

**Authors:** P J Commerford

**Affiliations:** Editor-in-Chief

It is a great privilege to be able to edit this publication as it strives to record the development of research and services aimed at alleviating cardiovascular diseases prevalent on the continent. I am delighted that so many original research articles, reviews and case reports from Africa are being submitted for publication.

In this regard, I do need to comment on the fact that there is another important aspect and responsibility of which all aspirant authors and researchers need to be cognisant. This is the need to be prepared to accept the responsibility of reviewing the work of peers and advising about suitability for publication. Authors often complain (legitimately) about delays in reviews and the publication of their work but are themselves reluctant to accept the invitation to review. My view is that it is essential that if we wish to continue to be an African journal, we need a core body of reviewers who understand Africa and the constraints of practice and research in Africa, and who are prepared to reviewsubmissions on the basis of their own local experience. I appeal to all of you, our readers, to accept requests for reviews when requested. If you are not already listed as a reviewer and are prepared to be a reviewer, please submit your name, qualifications and e-mail details to me, with your preferred specialities and area of review to patrick.commerford@uct.ac.za.

In this issue, Amadi and colleagues (page 106) document, in a survey of long-distance male bus drivers from Lagos in Nigeria, the frequency of risk factors for cardiac disease. This information is not surprising, given information from other parts of the world, but hopefully may be helpful in guiding employers, unions and individuals in Africa towards guiding employees regarding adoption of healthier lifestyles.

An issue that is often raised in discussions of clinical researchis whether such research translates into clinical benefit forpatients, and patients and researchers need an answer to that question. Prendergast and co-authors address that on page 98 of this issue. Their study demonstrates that participation in clinical research on rheumatic heart disease (RHD) can have a positive impact on patient management. Furthermore, REMEDY has led to increased patient awareness and improved healthcareworkers’ knowledge and efficiency in caring for RHD patients. The researchers are to be commended for demonstrating that research has had immediate positive results for patients participating in the research.

Little is known about the frequency and management of disturbances of cardiac rhythm in Africa and the report fromTalle and colleagues (page 115), which highlights this dearthof information and lack of clinical services, is timely anduseful. The information supplied by Kaduka and colleagues on stroke patterns in Kenya (page 68) is important and helpful. Unexpected observations include the preponderance of women affected by cerebrovascular disease and that cigarette smoking was the second most common risk factor.

In contrast to the issues above, which reflect many of the unresolved clinical issues of medicine and cardiology in Africa,it is a pleasure to be able to publish the work of Venter and colleagues (page 122), which reviews the molecular and cellular basis of cardiac disease.

